# Gestational diabetes mellitus and type 2 diabetes: same disease in a different moment of life? Maybe not

**DOI:** 10.1590/2359-3997000000276

**Published:** 2022-05-01

**Authors:** Lenita Zajdenverg, Carlos Antonio Negrato

**Affiliations:** 1 Departamento de Clínica Médica Universidade Federal do Rio de Janeiro Rio de Janeiro RJ Brasil Serviço de Nutrologia e Diabetes, Departamento de Clínica Médica, Universidade Federal do Rio de Janeiro (UFRJ), Rio de Janeiro, RJ, Brasil; 2 Associação dos Diabéticos de Bauru Bauru SP Brasil Associação dos Diabéticos de Bauru, Bauru, SP, Brasil

Gestational diabetes mellitus (GDM) is a medical condition that has motivated many debates in the last decades regarding its etiology, pathophysiology, diagnosis, treatment and long-term consequences to the mother and to the fetus.

Women who develop GDM present a metabolic condition similar to that found in type 2 diabetes (T2D) characterized by insulin resistance associated with inadequate insulin secretion (1). Due to similar pathophysiologic mechanisms found between T2D and GDM, there is a great interest in finding markers that will lead to the understanding of a possible common origin to both diseases. Women with GDM also present an inflammatory state that, together with insulin resistance can alter placental gene transcription and many features of fetal programming, that can lead to the development of several metabolic diseases later in life such as glucose intolerance, metabolic syndrome and also a high risk of presenting cardiovascular disease. Women with GDM have sevenfold higher risk of having T2D in the future (2).

Identifying risk markers for the development of GDM or for poor perinatal outcomes will allow the implementation of precocious preventive or therapeutic interventions. Recently, several biomarkers have been evaluated in order to establish this possible relationship such as cord blood adiponectin, C-reactive protein (CRP), advanced glycation end products (AGEs) and a variety of genetic polymorphisms.

Adiponectin exhibits an anti-inflammatory action and may potentially play a protective role in the development of GDM and T2D. Data regarding the relationship between cord blood levels of adiponectin, newborns birth weight and children adiposity are contradictory, with some studies finding a positive correlation (3) and others not showing any correlation (4).

In the present issue of the “*Archives of Endocrinology and Metabolism*” in a study conducted by Aramesh and cols. in Iran, 52 women with GDM and 52 with normal glucose tolerance (NGT) were evaluated regarding fetal anthropometric parameters, cord blood adiponectin and CRP. It was found that adiponectin levels were higher in the presence of GDM and was also associated with higher birth weight and later gestational ages. The levels of CRP were not different between the two groups (5). This finding contrasts with most studies associating low levels of adiponectin and increased levels of CRP with the risk of progression to T2D (6). Also published in this issue of “*Archives of Endocrinology and Metabolism*”, Lobo Jr. and cols. performed a study with 442 Euro-Brazilian women of which 225 had GDM and 217 presented NGT. Their study had the objective of evaluating the use of serum AGEs as a screening tool for GDM (7). It is well known that AGEs concentrations are associated with several diseases including type 1 and type 2 diabetes mainly in the presence of diabetes-related chronic complications (8). It is supposed that high oxidative stress conditions are associated with inflammation and the presence of diabetes. According to the authors, in their study, women with GDM had a good glycemic control which could have influenced the final results. They did not find different AGEs concentrations in GDM, possibly due to the mild severity and short duration of hyperglycemia. It is possible that in this environment they do not generate enough serum AGEs to make it possible discriminate between GDM and NGT groups (7).

The third study published in this issue refers to a cohort of Euro-Brazilian Caucasians formed by 252 patients, 127 with GDM and 125 with NGT. Anghebem-Oliveira and cols. evaluated the polymorphisms of several genetic variants that are associated with T2D. The authors studied gene polymorphisms T2D-related such as fat mass and obesity-associated (FTO), leptin receptor (LEPR), peroxisome proliferator-activated receptor gamma (PPARγ), and transcription factor 7-like 2 (TCF7L2) (9). These polymorphisms are related to food intake, energy balance, appetite regulation, gene expression transcription, glucose and lipids metabolism, inflammation and proliferation of pancreatic beta cells. Some of TCF7L2 polymorphisms have been found to be associated with GDM in other populations (10-13). The authors have found no relation between these polymorphisms with GDM in this Brazilian studied population (9).

The association between previous diagnoses of GDM with high risk of developing T2D is well known (2). Moreover, the screening of diabetes during pregnancy can lead to the discovery of an undiagnosed patient with T2D.

Although the reduction in insulin sensitivity and impaired insulin secretion occur similarly in cases of GDM and T2D, the dysglycemia found in GDM is generally transitory and disappears after delivery. However, the evaluation of non-pregnant women with glucose intolerance that participated in the Diabetes Prevention Program Outcomes Study, followed for ten years, has shown that those that had a history of GDM presented an increased risk of developing T2D when compared to those without previous GDM; this finding was independent of age and BMI. Interestingly, the reduction in the progression to T2D was found only in the group that had had GDM and were treated with metformin (14).

It is also important to note that although hyperglycemia that is first diagnosed during pregnancy is named GDM, it is known that a small percentage of these patients will require further reclassification. The most common types of monogenetic diabetes are frequently diagnosed by the first time during antenatal follow-up (15). Recently, Anghebem-Oliveira and cols., which did not find the presence of the gen polymorphisms associated with T2D and obesity, has found in this same Brazilian population of pregnant women a higher frequency of carriers of the polimorphism of the C allele of rs780094 from the glucokinase regulatory protein (GCKR) gen in the group of women with GDM (16). The inclusion, even of a small group of pregnant women probably with monogenetic diabetes, that is not associated with the same pathophysiologic mechanisms of T2D, can interfere with the interpretation of results in studies with a small number of patients.

Dysglycemia found in pregnancy can have different origins and complexities that may not be related to T2D, as proposed by the three manuscripts that were published in this periodic. Further studies are still required to find possible markers for GDM and T2D in order to discover the link that may exist or not between the two conditions.
